# Heterochromatin protein 1α interacts with parallel RNA and DNA G-quadruplexes

**DOI:** 10.1093/nar/gkz1138

**Published:** 2019-12-04

**Authors:** Ruby J Roach, Miguel Garavís, Carlos González, Geoffrey B Jameson, Vyacheslav V Filichev, Tracy K Hale

**Affiliations:** 1 School of Fundamental Sciences, Massey University, Private Bag 11–222, Palmerston North 4442, New Zealand; 2 Instituto de Química Física ‘Rocasolano’, CSIC, Serrano 119, 28006 Madrid, Spain; 3 Maurice Wilkins Centre, Private Bag 92019, Auckland, New Zealand

## Abstract

The eukaryotic genome is functionally organized into domains of transcriptionally active euchromatin and domains of highly compact transcriptionally silent heterochromatin. Heterochromatin is constitutively assembled at repetitive elements that include the telomeres and centromeres. The histone code model proposes that HP1α forms and maintains these domains of heterochromatin through the interaction of its chromodomain with trimethylated lysine 9 of histone 3, although this interaction is not the sole determinant. We show here that the unstructured hinge domain, necessary for the targeting of HP1α to constitutive heterochromatin, recognizes parallel G-quadruplex (G4) assemblies formed by the TElomeric Repeat-containing RNA (TERRA) transcribed from the telomere. This provides a mechanism by which TERRA can lead to the enrichment of HP1α at telomeres to maintain heterochromatin. Furthermore, we show that HP1α binds with a faster association rate to DNA G4s of parallel topology compared to antiparallel G4s that bind slowly or not at all. Such G4–DNAs are found in the regulatory regions of several oncogenes. This implicates specific non-canonical nucleic acid structures as determinants of HP1α function and thus RNA and DNA G4s need to be considered as contributors to chromatin domain organization and the epigenome.

## INTRODUCTION

Within the confines of the nucleus, genomic DNA is packaged with histone proteins to create highly folded yet dynamic chromatin fibres. At the most basic level DNA is wrapped 1.67 times around an octamer of four core histones to form a nucleosome (1). Arrays of nucleosomes undergo further folding to form a more condensed fibre. These chromatin fibres are further partitioned by architectural proteins into functionally distinct domains of transcriptionally active euchromatin and highly condensed transcriptionally silent heterochromatin, thereby ensuring appropriate patterns of gene expression and genomic stability (2,3).

**Figure 1.**
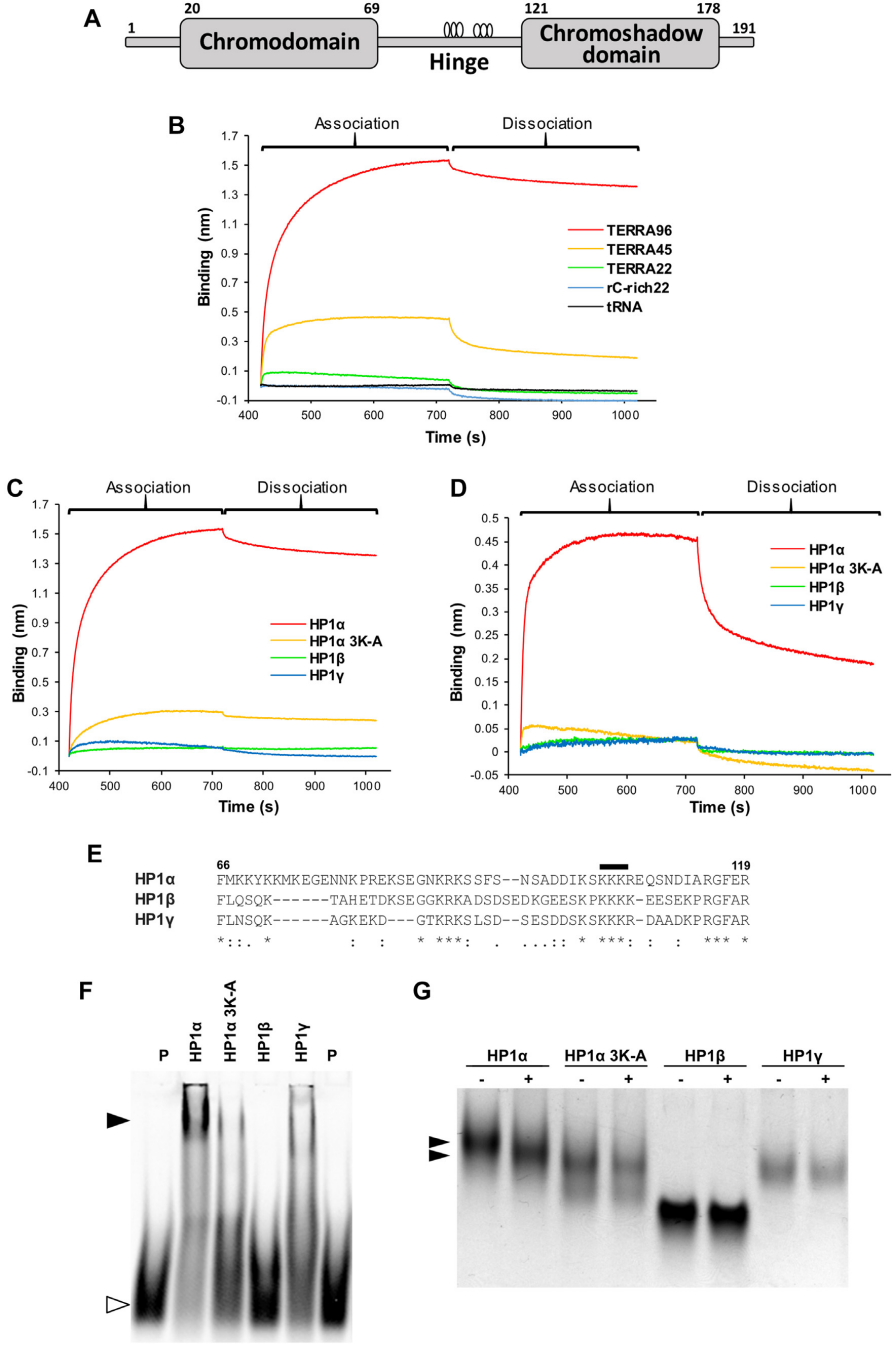
HP1α binds TERRA through a basic lysine patch in the hinge domain of HP1α only. (**A**) Schematic diagram showing the domain structure of mammalian HP1. The chromodomain and chromoshadow domain are linked by the hinge domain where the open circles indicate the location of two charged patches at residues 89-91 and 104-106. Residue numbers for HP1α are shown above. (**B**) Biolayer interferometry (BLI) analysis of immobilized HP1α binding to either TERRA96, TERRA45, TERRA22 or the controls, tRNA and rC-rich22. (**C**) BLI analysis of TERRA96 binding to either of the three HP1 paralogs (α, β, γ) or the HP1α 3K-A mutant. (**D**) BLI analysis of TERRA45 binding to either of the three HP1 paralogs or HP1α 3K-A. (**E**) Alignment of the hinge domains of HP1 paralogs. Black line indicates the lysine residues (104–106) mutated to alanine in HP1α 3K-A. The numbers refer to the amino acid positions of the first and last residues in the hinge sequence in relation to the amino acid sequence of HP1α. An asterisk (*) indicates a fully conserved residue. A colon (:) indicates conservation of a residue with strongly similar properties. A period (.) indicates conservation of a weakly similar residue. (**F**) Electrophoretic mobility shift analysis (EMSA) of TAMRA-labeled TERRA45 (TAM-TERRA45) in the absence (P) or presence of a 20-fold molar excess of the indicated HP1 proteins. Open arrow head denotes unbound TAM-TERRA45 probe, closed arrowhead denotes complex. (**G**) The HP1 paralogs and HP1α 3K-A, in solution with or without addition of TERRA45, separated by native PAGE. Arrows denote the change in migration of HP1α +/- TERRA45.

Members of the Heterochromatin Protein 1 (HP1) family are essential architectural proteins that establish and maintain heterochromatin (2,4,5). Mammalian cells contain three HP1 paralogs (α, β and γ) located on different chromosomes. HP1 consists of a conserved N-terminal chromodomain that binds histone H3 methylated on lysine 9 and a structurally related C-terminal chromoshadow domain that dimerizes and provides an interface for recruiting an array of proteins (Figure 1A). These domains are connected by a less conserved flexible hinge domain; also present are short unstructured N- and C-terminal extensions (6). The non-redundant functions of these highly conserved proteins that have emerged, and are reflected in their differing nuclear distributions, establish the need to identify the interactions that regulate and fine tune their individual functions within chromatin (7–9).

Constitutive heterochromatin, concentrated in pericentromeric and telomeric regions, is defined by the presence of HP1α and histone H3 trimethylated on lysine 9 (H3K9me3) (10–12). Although currently the paradigm of the histone-code, the interaction between the HP1α chromodomain and H3K9me3 (10,13) is not solely responsible for targeting HP1α to these condensed chromatin domains. Recent studies have demonstrated the requirement of the HP1α hinge domain, as well as RNA, in targeting HP1α to and retaining it on heterochromatin (14–16). *In vitro* the hinge domain binds nucleic acids with a preference for RNA, within this hinge region three basic residues (104–106) are required as their mutation to alanine disrupts this interaction with RNA (14,16). The establishment of a repressive chromatin environment on the pericentromeric and telomeric repetitive sequences is also dependent on non-coding repetitive RNAs that are transcribed from these sequences (17). Notably, HP1α can interact with TElomeric Repeat-containing RNA (TERRA) and major satellite repeat RNA, which are transcribed from the telomeric and pericentromeric regions, respectively (18–21). The interaction of HP1α with major satellite repeat RNA is mediated by the hinge domain and this interaction is responsible for the *de novo* targeting of HP1α to pericentromeric heterochromatin (21). This raises the question of how the HP1α hinge recognizes non-coding RNAs as previous *in vitro* binding studies suggest there is a degree of RNA structural specificity to this interaction (15,16).

Concomitantly, the maintenance of telomeric heterochromatin is essential to genome integrity as it protects the ends of chromosomes from degradation and the DNA damage response machinery (22). In mammals, telomeres comprise of highly conserved tandem repeats of (TTAGGG)_n_ terminating in a 3′ overhang (23). Transcription of these repeats from subtelomeric promoters results in TERRA transcripts of heterogeneous lengths with the majority consisting of UUAGGG tracts that are <400 bases (24). These guanine-rich transcripts form guanine-quadruplex (G4) structures by the association of four guanines bound through Hoogsteen hydrogen bonding (25–28). While it has been known for some time that DNA and RNA can form non-B structures *in vitro*, only recently has it been demonstrated that G4 structures, as well as intercalated C-rich motifs (i-motifs) that form in the complementary C-rich strands, exist *in vivo* and contribute to genome function (29–32). The presence of TERRA within the telomere nucleoprotein complex is proposed to act as a docking site for proteins involved in telomere function (17,24,33). One such protein is HP1α, whose interaction with TERRA is associated with its role in telomeric heterochromatin formation (18,24,34). So far, the binding of two telomere-associated proteins, Heterogeneous Nuclear Ribonucleoprotein A1 (hnRNPA1) and the TLS/FUS oncoprotein, to TERRA has been shown to be dependent on the presence of G4 structures (35,36).

To identify if the interaction of HP1α with RNA is structure dependent, we investigate whether HP1α by itself recognizes G4 structures formed by TERRA. Our findings demonstrate that the hinge region of HP1α can associate not only with TERRA G4 structures but also with G4 DNA structures, in both cases with a preference for G4s of parallel topology, whereas antiparallel G4s bind very slowly or not at all. This suggests that parallel G4 structures may play a role in how HP1α interacts with the chromatin fibre.

## MATERIALS AND METHODS

### Oligonucleotides

The G4 and i-motif oligonucleotides, except for TERRA96, (Supplementary Table S1) used in this study were obtained from Integrated DNA Technologies. TERRA96 was suspended in 100 mM NaCl, 10 mM NaH_2_PO_4_/ Na_2_HPO_4_, pH 8, and has been previously described (37,38).

G4-forming oligonucleotides were annealed in 1× Interaction Buffer 1 (1× IB1:100 mM KCl, 50 mM NaCl, 20 mM NaH_2_PO_4_/Na_2_HPO_4_, pH 8) by heating to 90°C for 5 min, cooling gradually to room temperature, while i-motifs and double-stranded DNA were annealed in 1× Interaction Buffer 2 (1× IB2: 100 mM KCl, 50 mM NaCl, 20 mM NaH_2_PO_4_/ Na_2_HPO_4_, pH 7), by heating to 90°C for 5 min and gradually cooling to room temperature. Experiments with folded G4 oligonucleotides were performed with 1× IB1 while experiments with folded i-motif oligonucleotides and double stranded DNA used 1× IB2.

Oligonucleotides used in this study were also separated on 20% native PAGE to determine their oligomeric state (Supplementary Figure S1).

### Protein expression and purification

The coding regions for mouse HP1 were subcloned from pGEX2T, previously described in (39), into the expression vector pPROEX HTb. The vector expressing the his-tagged HP1α hinge domain 3K-A mutant, where three lysines at positions 104–106 were replaced with alanine, was created from the pPROEX HTb hisHP1α vector using standard methods.

For protein expression, the hexahistidine-HP1 expression vectors were transformed into *Escherichia coli* Rosetta II cells (Merck, Germany) and expression induced with 0.4 mM IPTG (isopropyl-β-D-thiogalactopyranoside). After lysis, hexahistidine-HP1 paralogs were purified using the AKTA Prime Plus (GE Healthcare, UK) chromatography system and a Ni-NTA IMAC column by elution with 500 mM imidazole, concentrated using Vivaspin 20 (5 kDa MWCO) ultrafiltration devices (GE Healthcare) before passing through a Superdex 75 10/300 GL (GE Healthcare) size exclusion chromatography column, and finally re-concentrated as performed previously.

### Native polyacrylamide gel electrophoresis

Folded oligonucleotides (0.15 nmols) were separated on a 20% polyacrylamide gel in 0.5× TBE/0.5× IB1 (45 mM Tris-borate, 1 mM EDTA, 50 mM KCl, 25 mM NaCl, 10 mM NaH_2_PO_4_/ Na_2_HPO_4_, pH 8, or pH 7 for i-motifs) at 4°C. The gel was rinsed with H_2_O, stained with 0.35% Stains-All (Merck) in 50% formamide for 15 min, de-stained in H_2_O, and then imaged.

For the analysis of complex formation, purified his-tagged HP1 proteins (0.2 nmols) mixed with 0.3 nmols TERRA45 in 1× IB1 were separated on a 5% polyacrylamide gel in 0.5× TBE/0.5× IB1 at 4°C. The gel was then stained with QC Colloidal Coomassie Stain (Bio-Rad, USA) for 18 h at room temperature, de-stained with H_2_O and imaged.

### Electrophoretic mobility shift assay

Binding reactions were performed in a final volume of 20 μl using 10 pmol of 3′ TAMRA-labeled TERRA45 (TAM-TERRA45, Supplementary Table S1) and 20-fold molar excess of purified hexahistidine-HP1α (or the paralogs) in a binding buffer containing 50 mM Tris/HCl pH 7.5, 100 mM KCl, 0.5 mM EDTA, 0.5 mM dithiothreitol, and 0.1 mg/ml acetylated bovine serum albumin (Merck). Unlabeled competitor oligonucleotides (folded in 1× IB) were added at a 5- and 50-fold molar excess over TAM-TERRA45. After incubation at room temperature for 1 h, reactions were loaded onto a 4% polyacrylamide gel containing 0.5× TBE (45 mM Tris-borate, 1 mM EDTA, pH 8.3) and 50 mM KCl. Electrophoresis was then performed in 0.5× TBE and 50 mM KCl at 10 V/cm for 1 h at 4°C. Gels were imaged using a FLA-5000 Fluorescent Image Analyser (Fujifilm) at 532 nm.

### Circular dichroism spectroscopy

Oligonucleotides were prepared at 5–10 μM in 1× IB. Circular dichroism (CD) spectra were recorded using a Chirascan instrument (Applied Photophysics Ltd, UK).

Three scans were gathered over wavelengths ranging from 200 to 350 nm in a 0.1-cm path length cell at the standard sensitivity, data pitch 1 nm, continuous scanning mode, response 0.25 s, and bandwidth 1 nm. 1× IB was scanned as a buffer sample and its spectrum subtracted from the average of three scans for each sample. Each CD spectrum was smoothed by averaging 10 neighbor points using software provided by Applied Photophysics Ltd.

### Biolayer interferometry

Biolayer interferometry (BLI) using a BLItz system (ForteBio, USA) was used to examine the binding of HP1 to a range of oligonucleotides as indicated at room temperature.

Ni-NTA biosensors (ForteBio) were hydrated with 1× IB, and 4 μl of 100 μg/ml his-tagged HP1α, HP1α 3K-A, HP1β or HP1γ was used to load the Ni-NTA biosensor for 5 min to reach ∼4 nm of signal. The biosensor was then washed with 1× IB. The association step was performed either using 500 nM RNA or DNA oligonucleotides prepared in 1× IB or just 1× IB (reference) for 5 min, then the dissociation step was performed using 1× IB for 5 min. Reference runs were subtracted from test runs to account for dissociation of protein. All oligonucleotides were tested for interaction with Ni-NTA biosensor tips prior to experiments. BLItz Pro 1.2 software was used for curve fitting and *K*_D_ calculations.

For TERRA96, TERRA45, TERRA22, c-myc2, src2, and src16, values of *K*_D_ were obtained by titrating five or more concentrations of oligonucleotide against HP1α.

### Cell culture and indirect immunofluorescence analyses

NIH3T3 cells (ATCC, USA) were routinely cultured in Dulbecco's Modified Eagle Medium (DMEM) (11995, ThermoFisher Scientific) supplemented with 10% calf serum and 1% penicillin/streptomycin (Gibco, ThermoFisher Scientific) at 37°C and 5% CO_2_.

Asynchronously growing mouse NIH3T3 fibroblasts were washed with 1× Dulbecco’s Phosphate-Buffered Saline (DPBS, ThermoFisher Scientific), then CSK buffer (10 mM Pipes-KOH, pH 7, 100 mM NaCl, 300 mM sucrose, 3 mM MgCl_2_, Roche cOmplete protease inhibitor), before permeabilization in 0.5% Triton X-100/CSK buffer for 5 min. Cells were washed again with CSK buffer, then incubated for 15 min at room temperature with 2.5 μM of the indicated oligonucleotides in CSK buffer containing 1 U/μl SUPERaseIn RNase Inhibitor (ThermoFisher Scientific). After further washing with CSK buffer, cells were fixed with 2% paraformaldehyde/1× DPBS and processed for indirect immunofluorescence using an HP1α antibody (#2616, Cell Signaling Technology, USA) and anti-rabbit Alexa 647 secondary antibody (ab150079, Abcam, UK). Cells were then mounted in Slowfade Diamond Antifade Mountant with DAPI (ThermoFisher Scientific) on a microscope slide and imaged using a Leica SP5 DM6000B Scanning Confocal Microscope equipped with 63×/1.40 objective lens. Probes were excited with 405 and 633 nm excitation lasers, running LAS X software (Leica, Germany). All samples were imaged under identical conditions and digitally processed for presentation with Affinity Designer v1.6.1 (Serif Ltd, UK).

## RESULTS

### HP1α hinge interacts with TERRA

To determine the ability of HP1α to interact with telomeric repeat RNA, biolayer interferometry (BLI) was performed where purified his-tagged HP1α was immobilized on nickel sensor tips and the tips then immersed in a K^+^ solution containing G-rich RNA of varying lengths: 96 nucleotides (TERRA96), 45 nucleotides (TERRA45) or 22 nucleotides (TERRA22) from the telomeric RNA UUAGGG repeat sequence (see Supplementary Table S1 for oligonucleotide sequences used in this study). Figure 1B shows that the binding of TERRA to HP1α increases with increasing length of the TERRA oligonucleotide. Importantly, HP1α did not interact with two other RNA species tested. No interaction was detected between immobilized HP1α and tRNA (Figure 1B) in agreement with previous *in vitro* findings (16), nor was binding observed between HP1α and a cytosine-rich RNA of 22 nucleotides (rC-rich22, Figure 1B). Using BLI, a dissociation constant (*K*_D_) of 2.5 nM was determined for the interaction between HP1α and TERRA96 (Table 1 and Supplementary Figure S2A), while the HP1α–TERRA45 and HP1α–TERRA22 interactions had a *K*_D_ of 74 and 940 nM, respectively (Table 1; Supplementary Figure S2B and C).

**Table 1.** Association and dissociation rates, and dissociation constants for oligonucleotides with HP1α from BLI measurements. The oligonucleotide sequences are listed in Supplementary Table S1 and the CD spectra of the oligonucleotides are shown in Figures 2B and 3B, and Supplementary Figure S6

Oligonucleotide	Topology^a^	*k*_on_ / 1000 (M^−1^s^−1^)	*k*_off_ × 1000 (s^−1^)	*K*_D_ (nM)
**RNA G4**				
TERRA96	p	159 (9)^b^	0.40 (4)	2.5 (3)
TERRA45	p	219 (8)	16.0 (4)	74.4 (3)
TERRA22	p	41.4 (3)	39.0 (23)	940 (60)
mutTERRA45	pf	no binding		
**DNA G4: variable loop length**				
Oligo A	p	183 (4)	15.9 (2)	86.8 (2)
Oligo B	p	121.7 (13)	8.42 (7)	69.1 (9)
Oligo C	p	306 (7)	23.9 (7)	78.2 (3)
Oligo D	p	100.1 (13)	5.27 (9)	52.6 (11)
Oligo E	p	67.3 (10)	2.95 (5)	43.8 (10)
Oligo F	p	107 (15)	6.62 (1)	61.7 (15)
Oligo G	ap	no binding		
**DNA G4: from genomic regulatory regions**				
kras	p	67.0 (4)	4.61 (4)	68.8 (7)
bcl2	p	39.7 (6)	3.72 (4)	93.5 (16)
src3	p	36.0 (5)	5.03 (4)	140 (2)
ckit2	p	39.0 (4)	7.74 (6)	199 (3)
c-myc2	p	74 (4)	45.5 (13)	610 (40)
src2	ap	17.0 (8)	6.9 (3)	410 (20)
src16	ap	2.0 (2.0)	2.7 (4)	1630 (1700)

^a^G4 topology: (p) denotes parallel topology, (ap) denotes antiparallel topology and (pf) partially folded.

^b^The error in the least significant digit is provided in the parenthesis.

When HP1β and HP1γ were immobilized on nickel sensor tips, and the tips then immersed in a K^+^ solution containing TERRA96 (Figure 1C) or TERRA45 (Figure 1D), no binding occurred with these HP1 paralogs and TERRA45 or TERRA96, in contrast to the highly stable complexes observed between HP1α and either of the TERRA molecules.

To test if the hinge domain of HP1α is required for this interaction, an HP1α hinge mutant 3K-A (Figure 1E) was immobilized on the sensor tip and tested for its binding to TERRA96 and TERRA45. The substitution of three lysines (residues 104-106) within the hinge domain of HP1α for alanine prevents its interaction with RNA and DNA (14,16) and, as shown in Figure 1C and D, also markedly reduces binding of TERRA96 and TERRA45, respectively. The contribution of the HP1α hinge domain in mediating the binding to TERRA45 was confirmed by electrophoretic mobility shift assay (EMSA, Figure 1F). While HP1α formed a complex with TAMRA-labeled TERRA45 (TAM-TERRA45; Supplementary Table S1), the mobility of TAM-TERRA45 was not impeded in the presence of HP1β, and only partially impeded in the presence of either HP1α 3K-A or HP1γ, reflecting the BLI results presented in Figure 1D.

When the HP1 proteins were mixed with TERRA45 in solution and separated by native PAGE (Figure 1G), the faster migration of the HP1α/TERRA45 mixture compared to HP1α alone indicates there is a change in the overall charge/shape of HP1α when complexed to TERRA45. Of note also, is the faster migration of the HP1α hinge mutant 3K-A compared to wild-type HP1α. The similar circular dichroism (CD) spectra for these proteins indicates this is not due to protein misfolding of the HP1α 3K-A mutant (Supplementary Figure S3).

### HP1α interacts with parallel G4 structures

**Figure 2.**
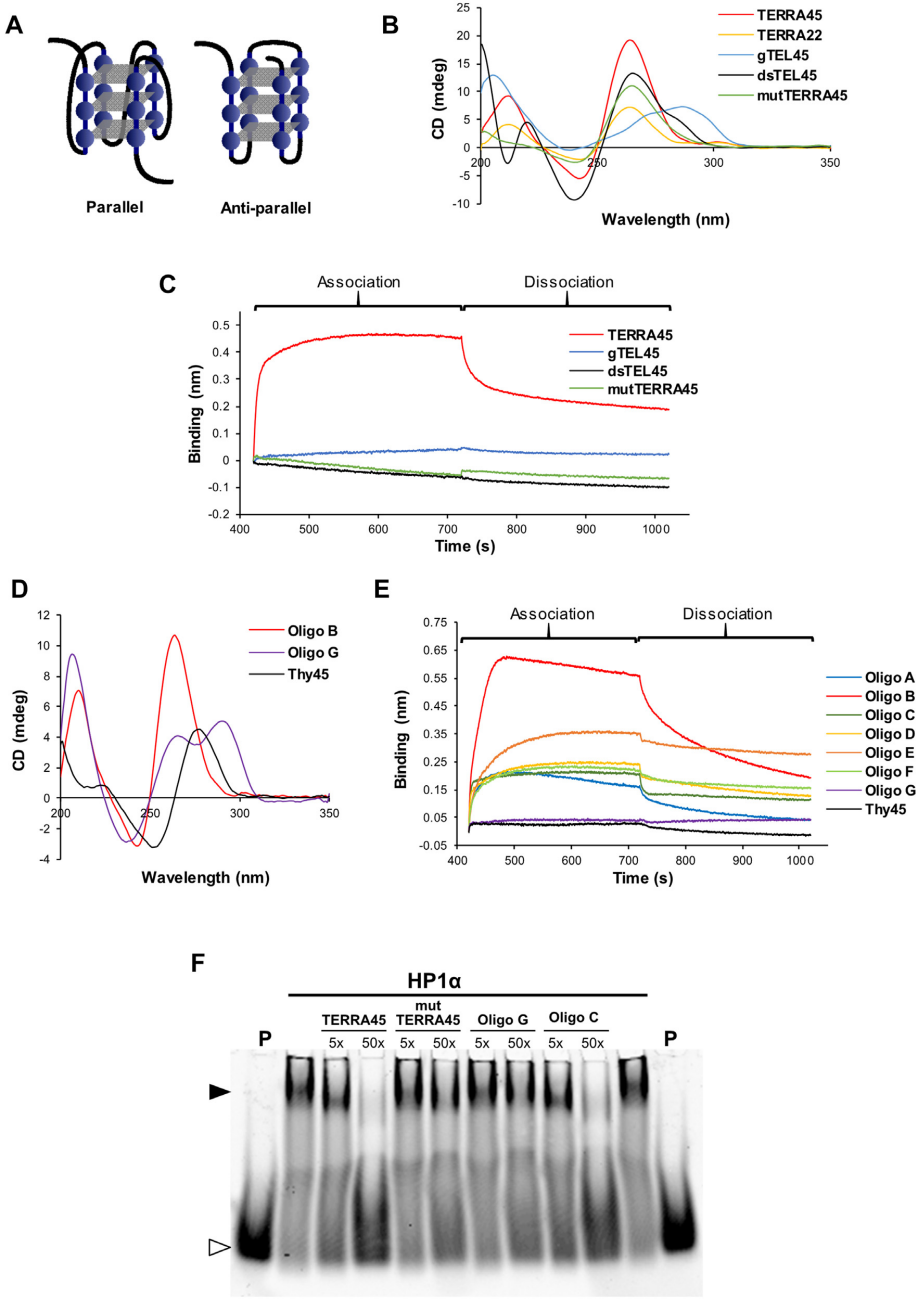
HP1α binds preferentially to G4s of parallel topology. (**A**) Schematic representation of a parallel and anti-parallel G4. Guanosines are represented as blue circles, individual G-tetrad as grey rectangles and loops as black lines. (**B**) Circular dichroism (CD) spectra of TERRA45, TERRA22, mutTERRA45, gTEL45, and telomeric duplex DNA (dsTEL45). (**C**) BLI analysis of HP1α binding to TERRA45, mutTERRA45, gTEL45 and dsTEL45. (**D**) CD spectra of Oligo B, Oligo G and Thy45. (**E**) BLI analysis of HP1α binding to oligonucleotides A-G with variant telomeric DNA repeat sequences or Thy45 DNA. (**F**) An EMSA competition assay showing HP1α binding to TAM-TERRA45 in the absence or presence of 5- and 50-fold molar excess of indicated oligonucleotides. (P) indicates TAM-TERRA45 probe only, open arrow head denotes unbound probe, closed arrowhead denotes complex.

CD spectroscopy (40) demonstrates that TERRA oligonucleotides form G4 species of parallel topology where all strands are in the parallel orientation with the homopolar stacking of G-quartets (Figure 2A) (37,38,41). The spectra of such G4 parallel structures have peaks with positive ellipticities at 210 and 265 nm and a peak with negative ellipticity at 240 nm (Figure 2B, group I according to classification proposed by Karsisiotis *et al* (42)). With the number of UUAGGG repeats, the TERRA96, TERRA45 and TERRA22 oligonucleotides have the potential to form four, two, and one G4 structure, respectively.

To test if the binding of HP1α to TERRA requires formation of the G4 structure, HP1α was immobilized on nickel sensor tips and then immersed in solutions of either TERRA45 or mutTERRA45, a 45-mer RNA in which four central GGG-tracts of TERRA45 were replaced with GUG (Supplementary Table S1). These substitutions result in a partial loss of G4 folding (Figure 2B) and a loss of HP1α binding (Figure 2C). Further supporting the requirement for G4 secondary structure, binding to HP1α was also reduced when TERRA45 was folded in a buffer containing 100 mM lithium chloride that hinders G4 formation (Supplementary Figure S4) (43).

Since TERRA as well as telomeric DNA can readily form G4 species, to determine if the specificity of the HP1α interaction is dependent on the nucleic acid forming a G4 assembly, gTEL45, a 45-nucleotide G-rich telomere repeat DNA oligonucleotide was tested for its interaction with HP1α (Figure 2C). However, no interaction was detected between HP1α and gTEL45. In contrast to TERRA45, this 45-mer telomeric DNA sequence adopts a different G4 topology according to its CD spectrum (Figure 2B). A peak with a positive ellipticity at 295 nm is a sign of heteropolar stacking between G-tetrads usually resulting in changes of strand orientation in loops of G4 species and formation of so-called anti-parallel G4 species (group II according to classification proposed by (42), Figure 2A). Also, unable to interact with HP1α was dsTEL45, a 45-nucleotide double-stranded DNA composed of telomere repeats (Figure 2B and C). HP1β and HP1γ, as well as HP1α 3K-A, were also tested for their ability to interact with the anti-parallel G4 formed in telomeric DNA (gTEL45); however, no binding was detected (Supplementary Figure S5). Overall, these data strongly suggest that HP1α interacts preferentially with parallel G4 structures.

To further check if it is G4 topology that determines the interaction between HP1α and G4s, we tested several DNA oligonucleotides derived from the 45-mer telomeric repeat in which the length of loop regions varied but the number of G-tracts (eight GGG motifs) was the same (Oligo A-G, see Supplementary Table S1). It is generally accepted that shortening of loops from three to one nucleotide can force G4s to adopt a parallel rather than anti-parallel topology (44). In all cases except Oligo G, these oligonucleotides formed G4s of parallel topology (group I), as observed in CD spectra, and interacted with immobilized HP1α in BLI (Figure 2D and E; Supplementary Figure S6). Oligo A, which had the shortest loops (1 nucleotide) in this series, formed a parallel G4, but had the highest detectable *K*_D_ among Oligos A-G (Table 1), indicating a weak interaction. Oligo B, with two nucleotides in the loops, existed in a monomer–dimer equilibrium as evidenced by native PAGE (Supplementary Figure S1B) and had a slightly better *K*_D_ than that of Oligo A. Oligos E, D and F with different distribution of one-nucleotide and three-nucleotide loops in the sequence formed parallel G4 species of different molecularity in solution (Supplementary Figure S1B), and exhibited noticeably slower *k*_off_ than Oligos A-C, which contributed to lower *K*_D_ values. In contrast, Oligo G had only one short loop in the middle of the sequence but exhibited a peak with positive ellipticity at 295 nm in its CD spectrum (group II, anti-parallel or hybrid G4) and did not bind to HP1α. In this regard, Oligo G behaves in a similar manner to gTEL45. Unstructured single-stranded Thy45, consisting of 45 thymidines, also did not interact with HP1α. Therefore, DNA topology and the length of loops in G4 assemblies are key in determining the interaction of HP1α with G4-forming single-stranded nucleic acids.

This preference for G4 topology was also confirmed by EMSA (Figure 2F) where binding of HP1α to fluorescently labeled TAM-TERRA45 was competed for by the presence of excess unlabeled TERRA45 and Oligo C, of parallel topology (Figure 2B and Supplementary Figure S6). However, the presence of the partially folded mutTERRA45 (Figure 2B) and Oligo G, an antiparallel DNA G4 (Figure 2D) did not alter HP1α/TAM-TERRA45 complex formation.

### Binding of HP1α to parallel G4 structures from genomic regulatory regions

**Figure 3.**
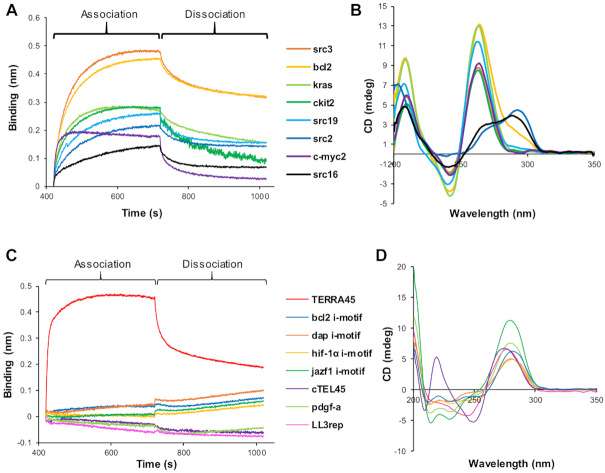
HP1α binds preferentially to endogenous non-B form DNA structures of parallel G4 topology. (**A**) BLI analysis of HP1α binding to genomic sequences that form G4 species. (**B**) CD spectra of sequences in (A). (**C**) BLI analysis of HP1α binding to i-motif forming oligonucleotides at pH 7, with binding of HP1α to TERRA45 for reference. (**D**) CD spectra of i-motif sequences used in (C) at pH 7.

The finding that HP1α interacts with DNA and RNA G4 structures of parallel topology suggests HP1α can recognize other parallel G4s identified in genomic DNA. BLI analysis was performed with HP1α immobilized on nickel sensor tips to test a selection of regulatory sequences of oncogenes known to form G4 species (Figure 3A). The oligonucleotides that had the fastest association with immobilized HP1α (*k*_on_, Table 1) were those shown by CD to have a parallel G4 topology (group I, Figure 3B). These include src3 located in the coding region of the *SRC* proto-oncogene and bcl2 from the promoter of the anti-apoptotic *BCL2* gene, which had a *k*_on_ of 3.60 × 10^4^ and 3.97 × 10^4^ s^−1^ M^−1^, respectively (Table 1) (45,46). The c-myc2 parallel G4 of 16 nucleotides, from the *MYC* proto-oncogene (47), is the shortest oligonucleotide, forming at least a G4 dimer according to the native PAGE (Supplementary Figure S1B), and had the fastest association with HP1α with a *k*_on_ of 7.43 × 10^4^ s^−1^ M^−1^ (Table 1). However, its fast release contributed to the weakest *K*_D_ detected among parallel G4s, which is also correlated with short loops present in this G4 (two one-nucleotide loops and one two-nucleotide loop). In comparison, G4-forming sequences from the *SRC* coding region that have features of an anti-parallel topology (group II, Figure 3B), src2 and src16, had the slowest association rates with immobilized HP1α (Figure 3A, Table 1), but probably because of long loops (4–7 nucleotides long) exhibited *k*_off_ values similar to the parallel G4s.

### HP1α does not bind the complementary C-rich strand

Recently, DNA sequences complementary to G-quadruplex forming sequences have been attracting much attention. The opposing C-rich strand derived from telomeres, centromeres and promoter regions are known to form i-motifs, a four-stranded structure in which two parallel duplexes intercalate with each other through C..H^+^..C base-pairs (48,49). When BLI was used to test the ability of six sequences known to partially form i-motifs at pH 7 (see Supplementary Table S1 and Figure S1C), including the telomeric C-rich strand (cTEL45), to bind immobilized HP1α (50), none of these assemblies interacted with HP1α (Figure 3C and D). We also tested one of the most stable i-motif structures at neutral pH, LL3rep, the mini-i-motif structure in which two C..H^+^..C stacks are capped with G:T:G:T tetrads (51), but again no interaction was detected with HP1α (Figure 3C). We note that i-motifs and mini-i-motifs have more similarity with anti-parallel rather than parallel G4s because of similarity in loops that connect the opposite columns in these assemblies.

### The nuclear spatial organization of HP1α is disrupted by competition with parallel G4s

**Figure 4.**
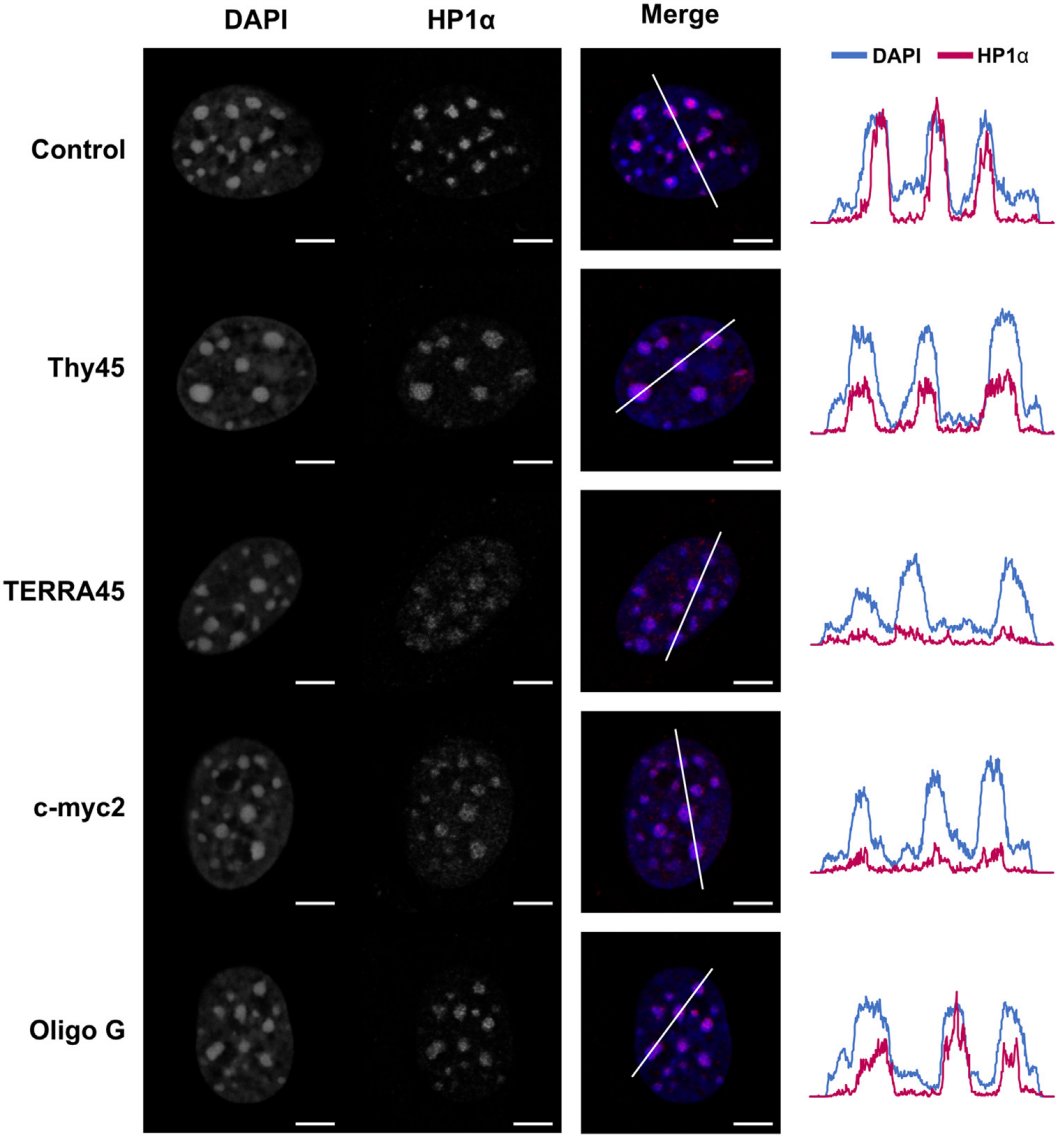
Parallel G4s compete for chromatin-bound HP1α *in situ*. Immunofluorescence analysis of asynchronous mouse NIH3T3 cells that were permeabilized before incubation with either 2.5 μM of Thy45, TERRA45, c-myc2, Oligo G or without oligonucleotide (control). After fixation, nuclear DNA was stained with DAPI and HP1α visualized by antibody detection. In merged images, DAPI (blue) and HP1α (red). Representative confocal sections are shown of individual cells (additional images of cells are presented in Supplementary Figure S7); scale bars: 5 μm. On the right-hand side are line plot profiles of fluorescent intensity showing the nuclear localization patterns of HP1α compared to DAPI-stained heterochromatin foci.

To test if parallel G4s could compete for chromatin-bound HP1α, an *in situ* competition assay was performed. Permeabilized unfixed mouse immortalized NIH3T3 fibroblasts were incubated with oligonucleotides of various topologies and the nuclear localization of HP1α detected by immunofluorescence (Figure 4). As expected, HP1α localizes to discrete DAPI-stained heterochromatin foci in nuclei of control NIH3T3 cells that were not incubated with an oligonucleotide. When cells were incubated with the unstructured Thy45 oligonucleotide or Oligo G, an anti-parallel G4 (Figure 2D), HP1α still localized to the heterochromatin foci although the level of HP1α retained within these loci was generally less than that of the control foci. In contrast, HP1α was no longer present in the DAPI-stained heterochromatin foci when cells were incubated with TERRA45 or c-myc2, which form RNA and DNA parallel G4 species (Figures 2B and 3B), respectively. Thus, it is parallel G4 species irrespective of DNA or RNA nature that were able to compete efficiently with heterochromatin for HP1α binding *in situ*.

## DISCUSSION

A major determinant in targeting HP1α to constitutive heterochromatin is the unstructured hinge domain. However, its role is the least understood of the three HP1 domains (8). Previously identified as a nucleic acid binding domain, we demonstrate here that the HP1α hinge has a strong preference for non-canonical G4 structures of parallel topology, regardless of whether formed of RNA or DNA, compared to anti-parallel DNA, i-motifs or canonical duplex DNA. The three lysine residues (104–106) within the hinge that are required for HP1α binding to chromatin and its targeting to heterochromatin are also necessary for the interaction with parallel G4 moieties (14,16). Although these residues are conserved among the HP1 paralogs (8), this interaction is unique to HP1α: as the other paralogs do not interact as strongly with the non-canonical structures tested suggesting that the interaction with the HP1α hinge is not solely determined by the charge of the three lysines. Supported by previous studies that show HP1β and HP1γ are less effective than HP1α at binding RNA and DNA, this differential binding of nucleic acids indicates a functional difference between the paralogs (9,16,18,52).

The main topological feature that differentiates parallel and anti-parallel G4 species is the position of loops that connect adjacent and opposite DNA or RNA strands in the structure (53,54). In parallel G4 species, propeller loops that link adjacent parallel strands do not interfere with G-tetrads. On the other hand, the lateral loops and diagonal loops that join anti-parallel strands are positioned above or below stacked G-tetrads, thus potentially interfering with possible interactions between G-tetrads and proteins. All parallel G4 species studied here exhibited fast association (*k*_on_) with HP1α in comparison with antiparallel G4s that bind very slowly or not at all. Therefore, from the kinetic perspective there is a clear preference for a specific G4 topology. Another non-canonical quadruplex structure, the i-motif, features diagonal loops and thus resembles anti-parallel rather than parallel G4 topology. This suggests that the loop orientation in the G4 structure is vital for its interaction with HP1α. Also required for this interaction is the positive charge patch within the hinge region of HP1α, presumably due to electrostatic interactions with the negative loops of G4s. Long loops in G4s contribute to slower *k*_off_ from HP1α as evident from the series of Oligos A-F. In addition, mutation of the three lysines in the hinge not only disrupts HP1α/G4 interactions but also increases the migration of the HP1α mutant during native PAGE relative to wild-type HP1α. This suggests that the mutation alters not only the charge but also the flexibility of the hinge to prevent the interaction with G4s. Presumably, the hinge becomes more structured upon binding the parallel G4 assembly and it will be of interest to determine how this interaction affects the behavior of the structured chromodomain and chromoshadow domains within chromatin.

There is a significant difference in *K*_D_ values of TERRA96 and TERRA45 with HP1α, being 2.5 and 74.4 nM, respectively. Since *k*_on_ values are similar, the difference in *K*_D_ lies in the very different *k*_off_ values (0.4 × 10^−3^ and 16 × 10^−3^ s^−1^, respectively). Unlike the BLI binding profile of TERRA45/96, the association of TERRA22 with HP1α is slow, and its release from the protein is faster than for TERRA45, which contributed to a high *K*_D_ value of 940 nM for TERRA22. Consistent with previously reported mass-spectrometry results (41), TERRA22 migrates as a dimer on native PAGE. Dimerization as well as multimerization of G4 species is typical for parallel topology (41,55) and bimolecular assemblies stacked via G-tetrad interfaces have been observed in NMR structures (56). In TERRA22, the formation of G4 structures implies the availability of three loops composed of three nucleotides in the individual G4 block. However, TERRA45 as well as TERRA96 form G4s with more loops per G4 and also longer loops that may involve one or two G-tracts that can contribute to the greater affinity of these folded oligonucleotides with HP1α in comparison with TERRA22. One should note, that TERRA96 is twice as long an assembly as TERRA45 and, unlike TERRA45, did not migrate under native PAGE. In long TERRA transcripts, G4s might be arranged as ‘beads-on-a-string’, in which each bead is an individual G4 or a two-block stacked G4 (57–60). Such long assemblies may interact with multiple HP1α molecules immobilized and densely packed on the tip in BLI leading to slow dissociation and low *K*_D_ values. Such a situation can be realized *in vivo* as HP1α is known to form higher order oligomers in a manner that is central to the formation and regulation of heterochromatin (61,62).

**Figure 5.**
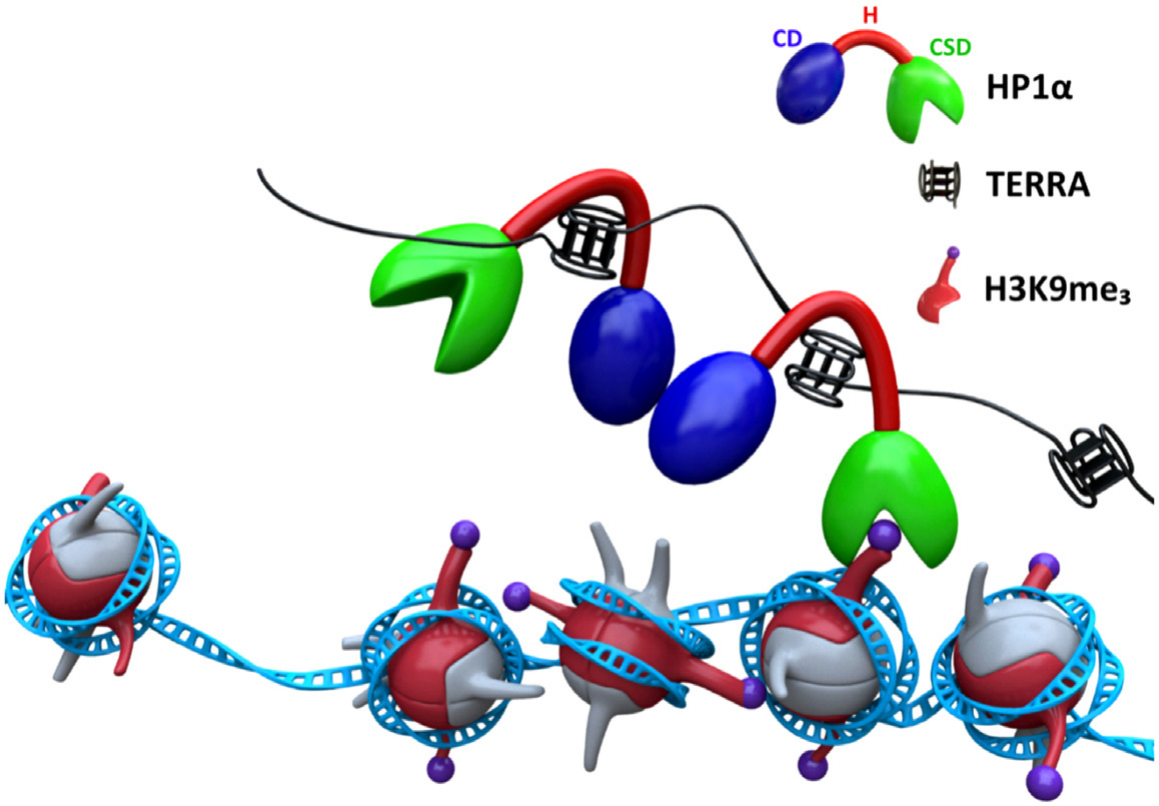
Model for TERRA-dependent enrichment of HP1α within telomeric heterochromatin. The ability of the HP1α hinge region to interact with G4s formed by TERRA ensures the targeting of HP1α to telomeres. HP1α dimerises through the chromoshadow domain (CSD) and this enrichment promotes the further interactions of HP1α within chromatin, including the interaction of its chromodomain (CD) with H3K9me3, to ensure the repetitive DNA at the telomere is maintained as heterochromatin.

The high affinity of HP1α for the G4 topology formed by TERRA96 provides a mechanism by which the non-coding transcript can promote heterochromatin formation at telomeres (Figure 5). The accumulation of TERRA in early G_1_ of the cell cycle would ensure an enrichment of HP1α at the telomere (63), promoting further multivalent interactions of HP1α with the chromatin fibre to reinforce the heterochromatinization of the telomere (21). That G4s of parallel topology play a role in nuclear organization of HP1α is demonstrated by the *in situ* competition assay, where the localization of HP1α within heterochromatin foci was disrupted by the presence of parallel G4s. An analogous mechanism appears to maintain pericentromeric heterochromatin where the RNA transcribed from major satellite repeat DNA mediates targeting of HP1α to this domain (21). Although these major satellite repeat transcripts are predicted to form RNA:DNA hybrids (64), the secondary structure of this repetitive RNA that provides specificity to its interaction with the HP1α hinge has yet to be identified.

To date, the formation of G4 species within genomic DNA has been associated with regions of euchromatin suggesting that heterochromatin suppresses G4 formation (65). In human cells, beyond the telomere, sequences predicted to form G4s are enriched in rDNA, several VNTRs and several single-copy genes, where they are found in promoters, 5′-UTR and the 5′-end of intron boundaries (66). Differential *k*_on_ values observed here for the interaction of HP1α with G4s of various topologies indicate that HP1α would preferentially interact with G4s formed during cell cycle progression of parallel rather than antiparallel topology (29). The demonstration that HP1α also interacts with parallel G4 assemblies known to form in the regulatory regions of several oncogenes raises the possibility that HP1α can interact with these parallel G4 assemblies within the DNA duplex *in vivo* (45,67–70). Therefore, their formation also has the potential to influence HP1α function in gene silencing, RNA processing and/or DNA repair (71–73).

In conclusion, our findings show that the HP1α paralog, a major determinant of constitutive heterochromatin, has a faster association rate with G4 species folded into a parallel rather than anti-parallel topology. Notably, an excess of these G4 assemblies can compete HP1α from heterochromatin *in situ*. Therefore, these G4 assemblies whether present in non-coding RNA or genomic DNA may serve as signposts for HP1α-mediated functions. These findings also provide further evidence that a ‘G4 genome’ plays a role in the regulation of the functional organization of the genome (66).

## DATA AVAILABILITY

The authors declare that the main data supporting the findings of this study are available within the article and its Extended data files. Extra data are available from the corresponding author upon request.

## Supplementary Material

gkz1138_Supplemental_File
