# Supplementary material for: Heterochromatin protein 1α interacts with parallel RNA and DNA G-quadruplexes
Source: Nucleic Acids Res. 2019 Dec 4;48(2):682–93. doi: 10.1093/nar/gkz1138 (PMC6954420; doi:10.1093/nar/gkz1138)
Supplement: gkz1138_Supplemental_File [file gkz1138_supplemental_file.pdf]

| Name | Sequence | Length | Topology |
|---|---|---|---|
| TERRA96 | rGrGrG rUrUrA rGrGrG rUrUrA rGrGrG rUrUrA rGrGrG rUrUrA<br>rGrGrG rUrUrA rGrGrG rUrUrA rGrGrG rUrUrA rGrGrG rUrUrA<br>rGrGrG rUrUrA rGrGrG rUrUrA rGrGrG rUrUrA rGrGrG rUrUrA<br>rGrGrG rUrUrA rGrGrG rUrUrA rGrGrG rUrUrA rGrGrG rUrUrA | 96 | p |
| TERRA45 | rGrGrG rUrUrA rGrGrG rUrUrA rGrGrG rUrUrA rGrGrG rUrUrA<br>rGrGrG rUrUrA rGrGrG rUrUrA rGrGrG rUrUrA rGrGrG | 45 | p |
| TAM-TERRA45 | rGrGrG rUrUrA rGrGrG rUrUrA rGrGrG rUrUrA rGrGrG rUrUrA<br>rGrGrG rUrUrA rGrGrG rUrUrA rGrGrG rUrUrA rGrGrG rU/TAM/ | 46 | p |
| mutTERRA45 | rGrGrG rUrUrA rGrUrG rUrUrA rGrUrG rUrUrA rGrGrG rUrUrA<br>rGrUrG rUrUrA rGrGrG rUrUrA rGrUrG rUrUrA rGrGrG | 45 | pf |
| TERRA22 | rArGrG rGrUrU rArGrG rGrUrU rArGrG rGrUrU rArGrG rG | 22 | p |
| rC-rich22 | rArCrC rCrArA rUrCrC rCrArA rUrCrC rCrArA rUrCrC rC | 22 | i |
| gTEL45 | GGG TTA GGG TTA GGG TTA GGG TTA GGG TTA GGG TTA GGG TTA GGG | 45 | ap |
| Thy45 | TTT TTT TTT TTT TTT TTT TTT TTT TTT TTT TTT TTT TTT TTT TTT | 45 | n/a |
| Oligo A | TGG GTG GGT GGG TGG GTG GGT GGG TGG GTG GGT | 33 | p |
| Oligo B | TGG GTT GGG TTG GGT TGG GTT GGG TTG GGT TGG GTT GGG T | 40 | p |
| Oligo C | TGG GTT AGG GTG GGT TAG GGT GGG TTA GGG TGG GTT AGG GT | 41 | p |
| Oligo D | TGG GTG GGT TAG GGT GGG TGG GTG GGT TAG GGT GGG T | 37 | p |
| Oligo E | TGG GTG GGT TAG GGT TAG GGT GGG TGG GTT AGG GTT AGG GT | 41 | p |
| Oligo F | TGG GTT AGG GTT AGG GTG GGT GGG TTA GGG TTA GGG TGG GT | 41 | p |
| Oligo G | TGG GTT AGG GTT AGG GTT AGG GTG GGT TAG GGT TAG GGT TAG GGT | 45 | ap |
| bcl2 | AGG GGC GGG CGC GGG AGG AAG GGG GCG GGA GCG GGG CTG | 39 | p |
| c-myc2 | GGG TGG GTA GGG TGG G | 16 | p |
| ckit2 | GGG CGG GCG CCA GGG AGG GG | 20 | p |
| kras | AGG GCG GTG TGG GAA GAG GGA AGA GGG GGA GG | 32 | p |
| src2 | GGG GCA GCT GGG TCG CTC GGG GAA CGG GG | 29 | ap |
| src3 | GGG AGG GAG GGC TGG GGG | 18 | p |
| src16 | GGG CTG CTG GGA CTG GTT GGG AGG GCA CTC TGG GG | 35 | ap |
| src19 | GGG TCC CCT GGG CCT GGG CGG GAG CGG GGA GGG G | 34 | p |
| LL3rep | TCG TTC CGT TTT TCG TTC CGT TTT TCG TTC CGT TTT TCG TTC CGT | 45 | i |
| DAP i-motif | CCC CCG CCC CCG CCC CCG CCC CCG CCC CC | 29 | i |
| hif-1α i-motif | CGC GCT CCC GCC CCC TCT CCC CTC CCC GCG C | 31 | i |
| pdgf-a | CCG CGC CCC TCC CCC GCC CCC GCC CCC GCC CCC CCC CCC CC | 41 | i |
| jazf1 i-motif | CCC CCC CCG CCC CCG CCC CCG CCC TCC CCC C | 31 | i |
| bcl2 i-motif | CAG CCC CGC TCC CGC CCC CTT CCT CCC GCG CCC GCC CCT | 39 | i |
| cTEL45 | CCC TAA CCC TAA CCC TAA CCC TAA CCC TAA CCC TAA CCC TAA CCC | 45 | i |

**Supplementary Table 1.** Sequences used in Biolayer Interferometry (BLI) and Circular Dichroism (CD) spectroscopy. G4 topology (as determined by CD spectroscopy) of each oligonucleotide listed where (a) denotes anti-parallel, (p) denotes parallel topology, (pf) denotes partially folded and (i) denotes i-motif.

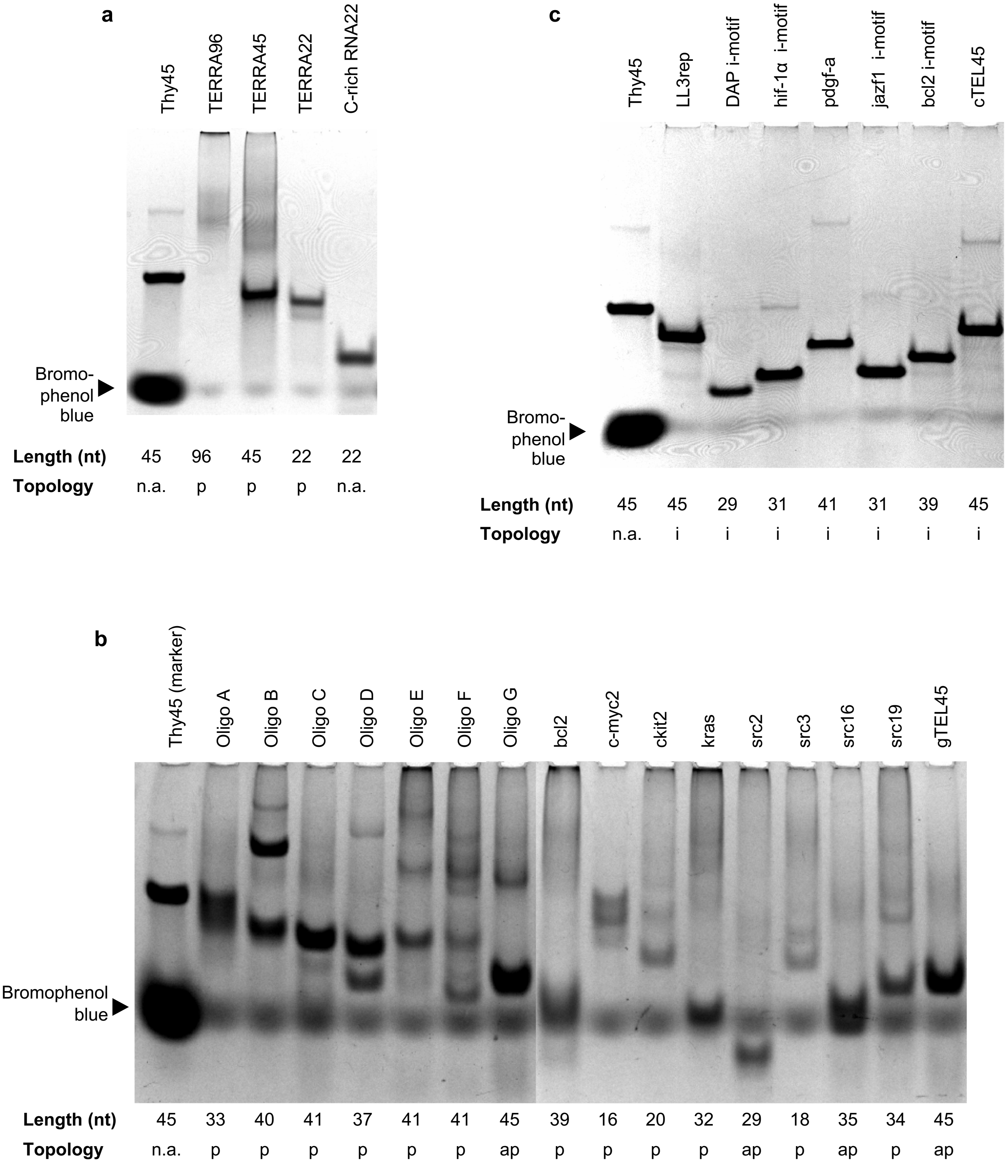


**Supplementary Figure 1.** Oligonucleotides separated by native 20% PAGE. A) Folded RNA oligonucleotides at pH 8. B) Folded DNA oligonucleotides at pH 8. C) i-Motif forming oligonucleotides at pH 7. Below the gel image is the length and DNA/RNA topology (as determined by CD spectroscopy) of each oligonucleotide listed where (a) denotes anti-parallel G4, (p) denotes parallel G4 topology and (i) denotes i-motif.

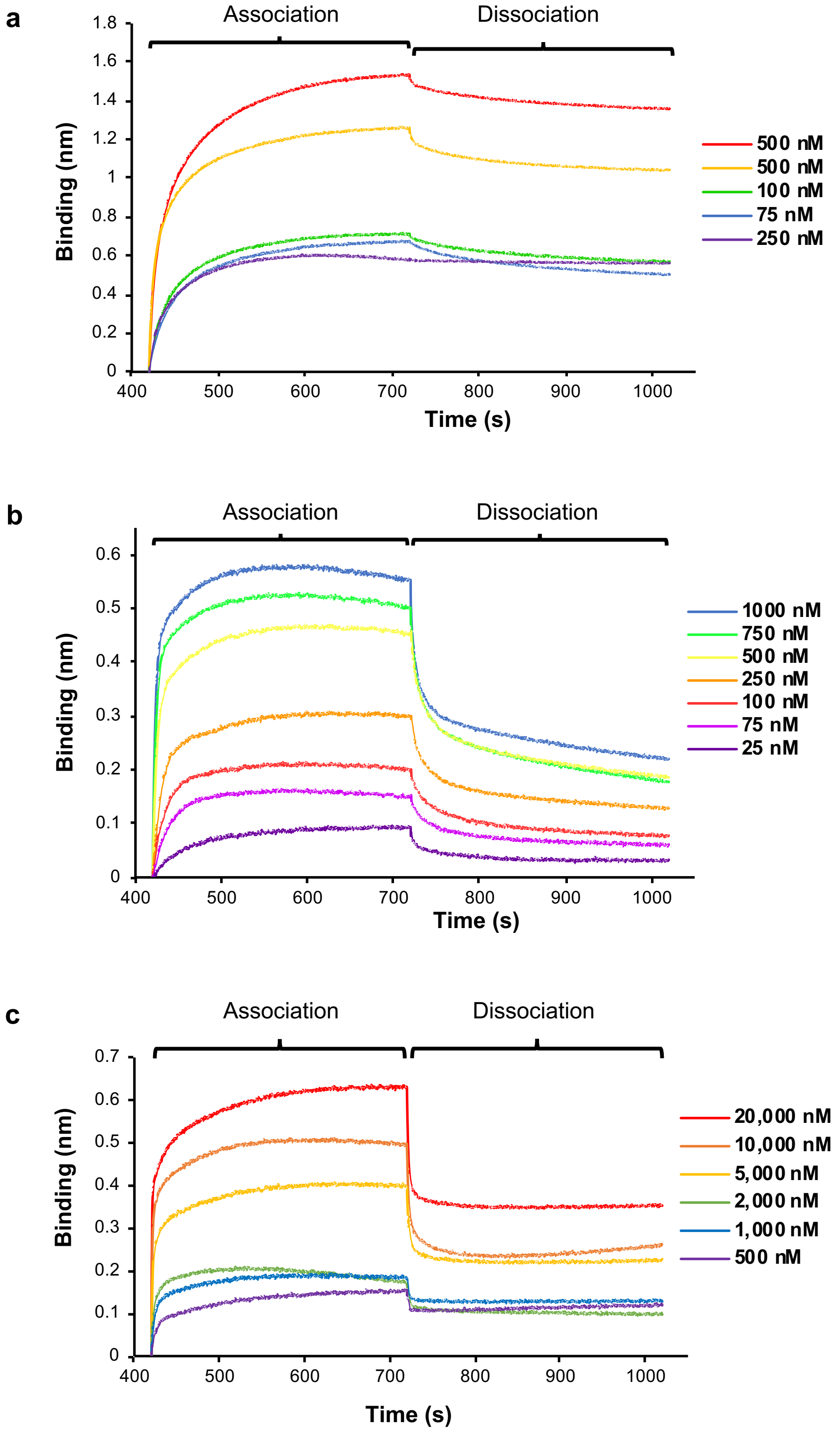


**Supplementary Figure 2.** The affinity of HP1α for TERRA determined by BLI. A) Binding and dissociation curves for HP1α with TERRA96 titrated from 75 nM to 500 nM. Association rate constant, $k_{on} = 1.59 \times 10^5$ ($\pm 0.09 \times 10^5$) $s^{-1}$ $M^{-1}$; dissociation rate constant, $k_{off} = 4.0 \times 10^{-4}$ ($\pm 0.4 \times 10^{-4}$) $s^{-1}$; equilibrium constant, $K_D = 2.5$ ($\pm$ 0.3) nM. B) Binding and dissociation curves for HP1α with TERRA45 titrated from 25 nM to 1,000 nM. Association rate constant, $k_{on} = 2.19 \times 10^5$ ($\pm 0.08 \times 10^5$) $s^{-1}$ $M^{-1}$; dissociation rate constant, $k_{off} = 1.60 \times 10^{-2}$ ($\pm$ $0.04 \times 10^{-2}$) $s^{-1}$; equilibrium constant, $K_D = 74.4$ ($\pm$ 0.4) nM. C) Binding and dissociation curves for HP1α with TERRA22 titrated from 500 nM to 20,000 nM. Association rate constant, $k_{on} = 4.10 \times 10^5$ ($\pm 0.08 \times 10^5$) $s^{-1}$ $M^{-1}$; dissociation rate constant, $k_{off} = 3.9 \times 10^{-2}$ ($\pm 0.2 \times 10^{-2}$) $s^{-1}$; equilibrium constant, $K_D = 940$ ($\pm$ 60) nM.

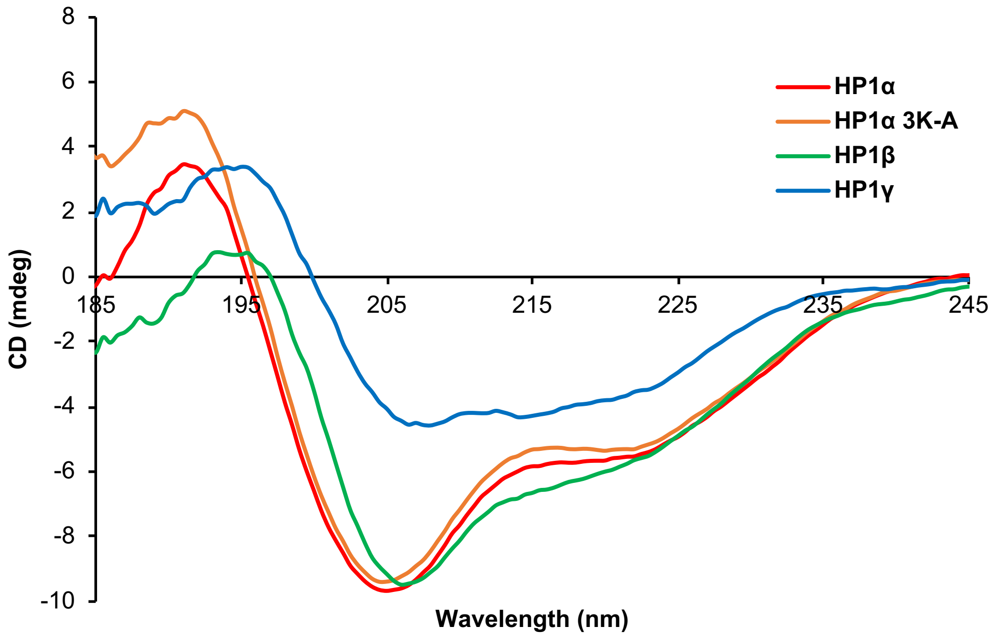


**Supplementary Figure 3.** CD spectra of HP1α, HP1β, HP1γ, and HP1α 3K-A at 1.4 mg/mL in 1x IB1 recorded in a 0.1 mm path length cuvette.

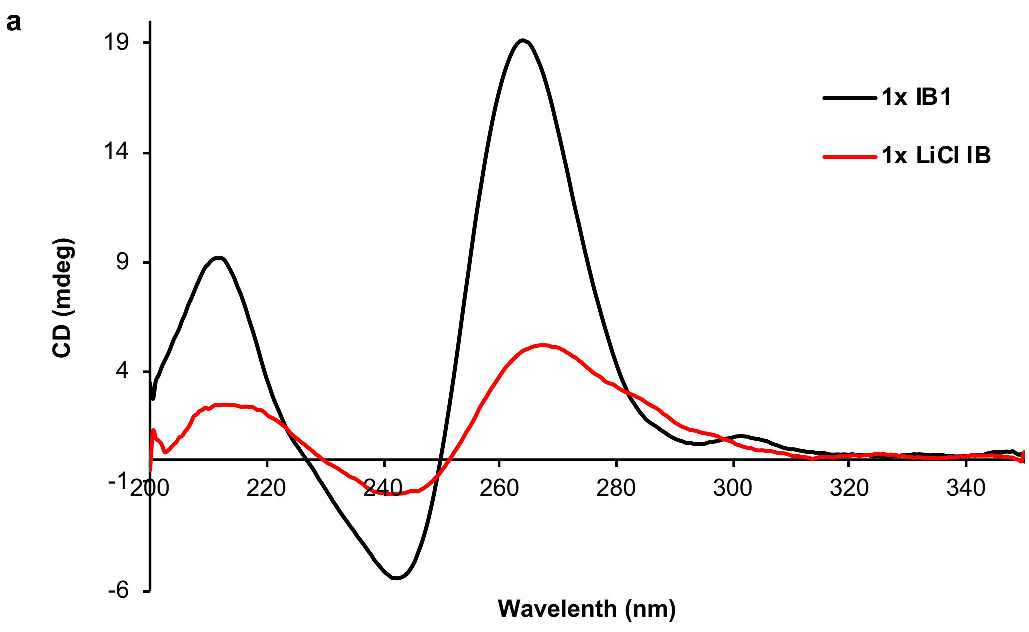


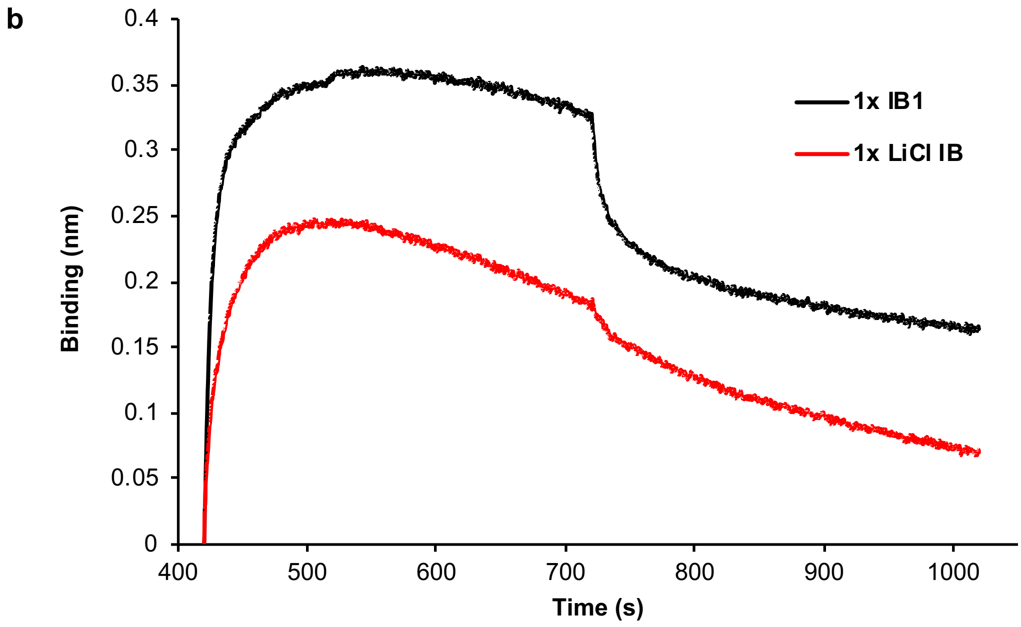


**Supplementary Figure 4.** TERRA45 in buffer containing lithium chloride reduces the interaction with HP1α. A) CD spectra of TERRA45 in 1x Interaction Buffer 1 (1x IB1: 100 mM KCl, 50 mM NaCl, 20 mM $NaH_2PO_4/Na_2HPO_4$, pH 8) and TERRA45 in 1x LiCl Interaction Buffer (1x LiCl IB: 100 mM LiCl, 70 mM Tris-HCl, pH 8) at 10 µM recorded in a 0.1 mm path length cuvette. B) BLI of TERRA45 binding to HP1α in 1x IB1 or in 1x LiCl IB.

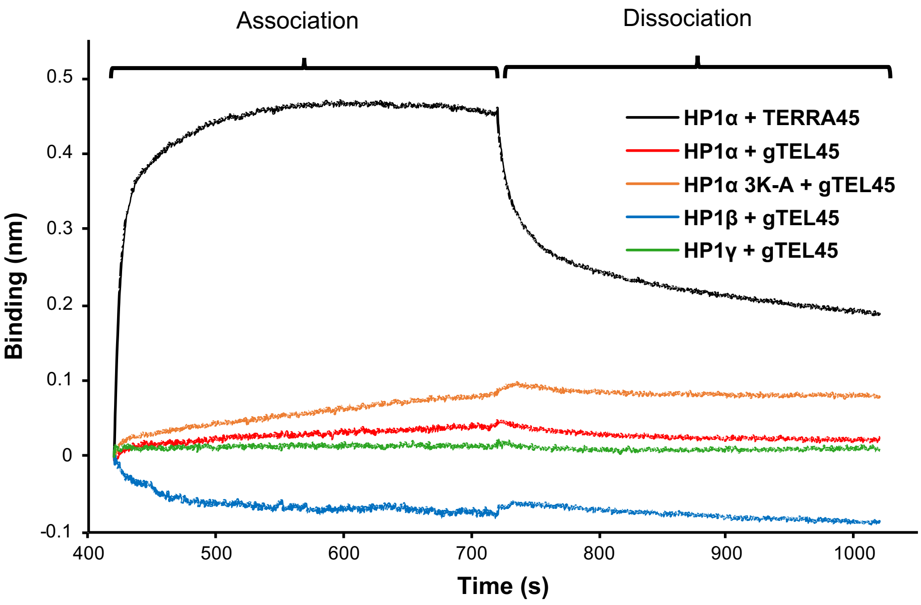


**Supplementary Figure 5.** BLI analysis to determine an interaction between either HP1β, HP1γ, or HP1α 3K-A and gTEL45, the telomeric DNA oligonucleotide that forms an anti-parallel G4. HP1α binding to TERRA45 is shown for reference.

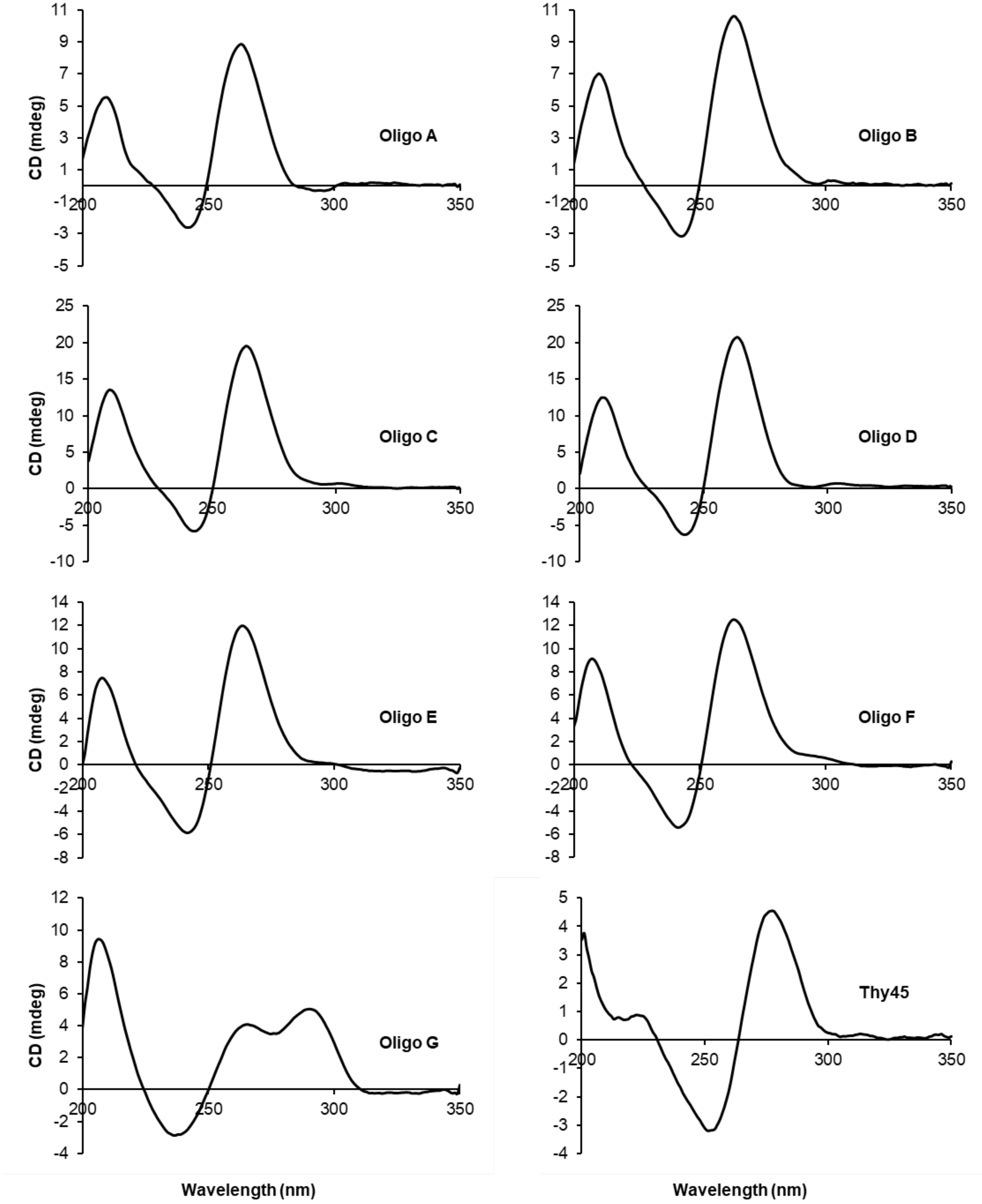


**Supplementary Figure 6.** CD spectra of DNA oligonucleotides, Oligo A-G, which have various telomeric DNA repeat sequences, and Thy45 DNA.

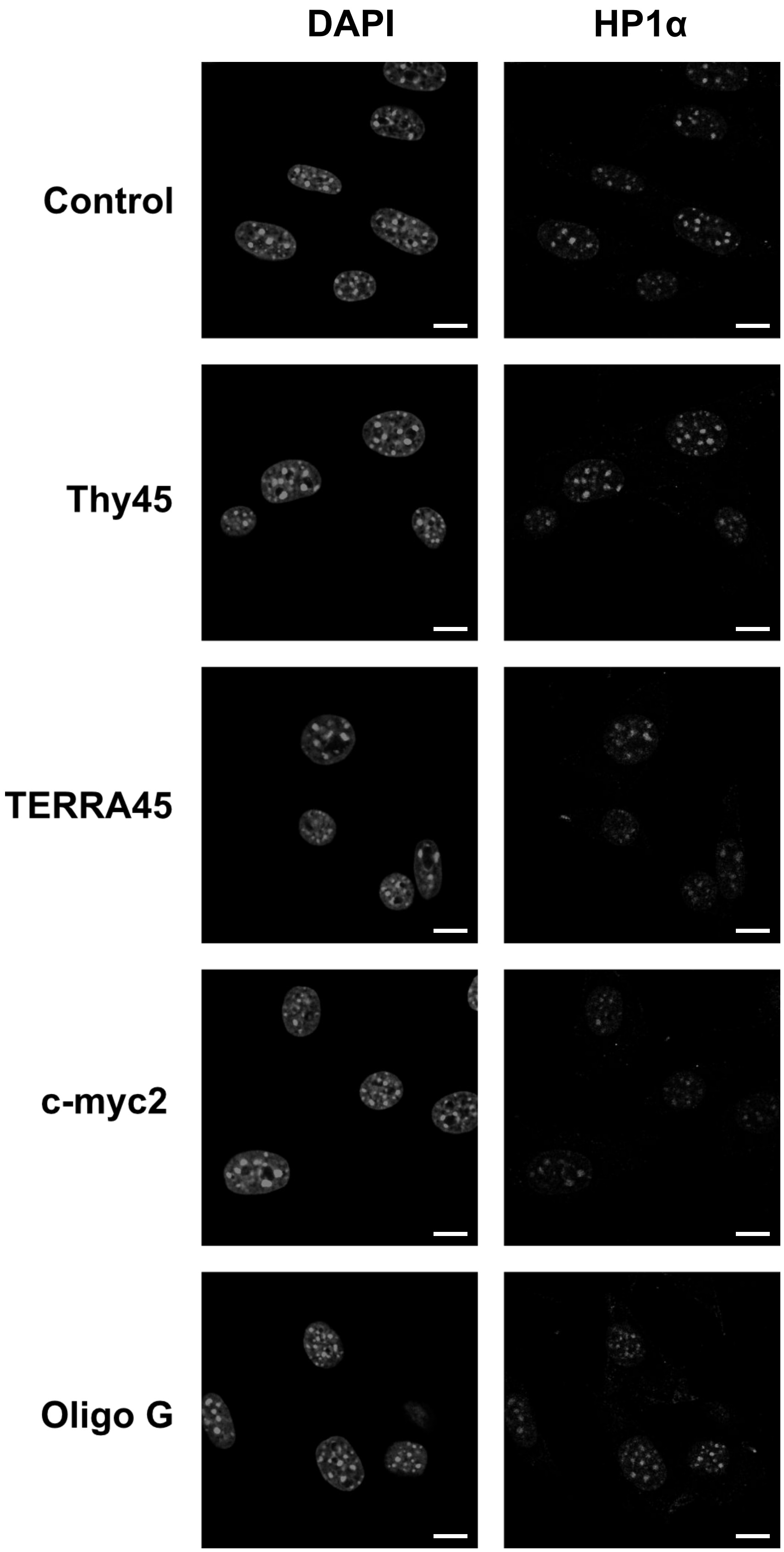


**Supplementary Figure 7.** Immunofluorescence analysis of asynchronous mouse NIH3T3 cells that were permeabilised before incubation with either 2.5 μM of Thy45, TERRA45, c-myc2, Oligo G or without oligonucleotide (control). After fixation, nuclear DNA was stained with DAPI and HP1α visualised by antibody detection. Representative confocal sections are shown. Scale bars, 10 μm.
